# Total Uterine Inversion Post Partum: Case Report and Management Strategies 

**Published:** 2018-12

**Authors:** Anthony Paulo Sunjaya, Andriana Kumala Dewi

**Affiliations:** 1 Department of Obstetrics and Gynecology, Faculty of Medicine, Tarumanagara University Jl. Letjen S. Parman, Jakarta, Indonesia; 2 Department of Obstetrics and Gynecology, Faculty of Medicine, Sumber Waras Hospital Tarumanagara University, Jl. Kyai Tapa, Jakarta, Indonesia

**Keywords:** Uterine Inversion, Atony, Manual Uterine Re-position, Balloon Tamponade

## Abstract

**Objective:** Total Uterine Inversion is a rare obstetric emergency that may lead to hypovolemia and eventually death. Its incidence varies between different populations and is reported to be between 1 in 2000 to 1 in 50,000 births. This article describes a case of acute total uterine inversion post-partum and review of its management strategies.

**Case report:** A primigravid 24 year old female 1.5 hours post-partum was referred to the emergency department from the community health center with total uterine inversion and hypovolemic shock. The patient had given birth vaginally with a midwife. We successfully performed manual reposition of the uterus and balloon tamponade was placed to stop the hemorrhage.

**Conclusion:** Total Uterine Inversion is a rare but potentially deadly complication post-vaginal delivery. Its low incidence leads to sparse experience among health professionals in managing this obstetrical emergency. Early fluid resuscitation, manual reposition and balloon tamponadeis essential in order to obtain the best prognosis.Further studies are required to determine the most optimal conservative and surgical management for uterine inversion.

## Introduction

Total Uterine Inversion is a rare obstetric emergency that may lead to hypovolemia and eventually death. Its incidence varies between different populations and is reported to be between 1-in-2000 to 1-in-50,000 births. Immediate active management of uterine inversion is recommended as the massive and often understimated blood loss is reported to be fatal in as much as 15% in some case series (1). However, its low incidence makes obstetricians experience regarding this condition scarce and the lack of studies resulted in the best management options not fully understood. Several therapeutic strategies have been previously reported including drugs, manual maneuvers and surgical interventions (2). This article describes a case of acute total uterine inversion post-partum and review of its management strategies. Informed consent was obtained from the patient prior to the publication of this article and available for review upon request.

## Case report

A primigravid 24 year old female 1.5 hours post-partum was referred to the emergency department from the community health center with total uterine inversion and hypovolemic shock. The patient had given birth vaginally with a midwife wherein during the third stage of labour while placental traction was performed to remove the placenta a large mass emerged through the vaginal passage with the placenta. Afterwards, the patient was reported to be bleeding profusely and soon lose consciousness. 

**Figure 1:**
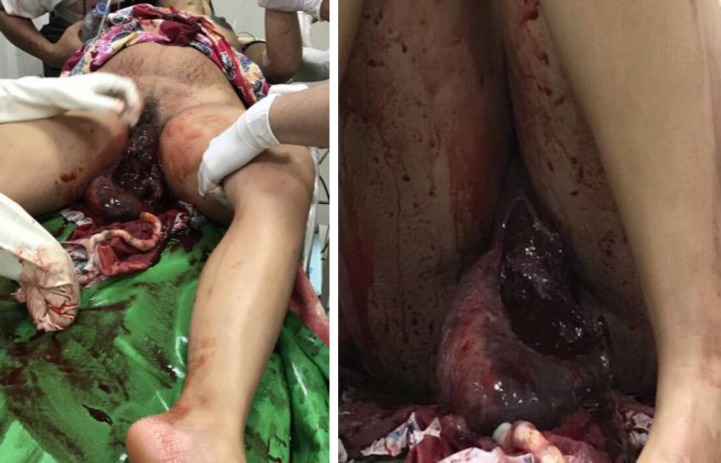
The inverted Uterus

On arrival at the emergency department, the patient was anemic and unresponsive with active vaginal bleeding. Physical examination revealed hypotension (blood pressure 80/60 mmHg), tachycardia (138 x/min) and tachypnea (26x/min). Conjunctiva was anemic with perioral cyanosis, cold & mottled extremities and prolonged capillary refill time (> 3 seconds). Inspection of the genitalia revealed total uterine inversion with perineal lacerations (Figure 1).

Immediate bloodwork at time of admission was performed which revealed anemia (haemoglobin 5.9 g/dL, hematocrit 17.4%, erythrocyte 2.07 million/uL) and leukocytosis (22.000/uL). Normal thrombocyte level (358.000/uL) was found.

The patient was treated by fluid resuscitation with colloids and crystalloids, blood transfusion and uterotonics. We successfully performed manual reposition of the uterus followed by internal bimanual compression for 15 minutes, however profuse hemorrhage was still observed due to uterine atony. Therefore balloon tamponade was placed to stop the hemorrhage and reduce risk of recurrence. The patient was then stabilized; no surgical management or hysterectomy was required. Perineal repair was subsequently performed once bleeding was managed.

Post-transfusion of 4 packed red cells, bloodwork was repeated which showed hemoglobin of 10 g/dL, hematocrit 29%, erythrocyte 3.66 million/uL, leucocyte 20.900/uL and thrombocyte 159.000/uL. Patient’s hemodynamic also became stable and regain consciousness. Three days post admission, patient recovered without complications. She was discharged with oral antibiotics and pain medication.

## Discussion

Uterine inversion is defined as the passage of the uterine fundus inferiorly into the uterine cavity and cervix, turning the uterus inside out. The fundus may be present in the uterine cavity (incomplete), through the cervical or even throught the vaginal introitus (1). Although a rare event, uterine inversion can occur in 2 distinct situations – post-partum and spontaneously. Ninety-five percent of all uterine inversions occur puerperal, non-puerperal uterine inversion is generally associated with exteriorization of uterine cavity tumors.Based on timing uterine inversion can be classified into 3 – acute (within 24 hours of delivery), subacute (> 24 hours but < 4 weeks) or chronic (> 1 month postpartum) (2).

The exact etiology of uterine inversion remains elusive, however strong traction of the umbilical cord particularly during the third stage of labour when the placenta is in a fundal location is hypothesize to be the most likely cause. Other factors include relaxed uterus, lower uterine segment and cervix; uterine fibroids; placenta accreta, particularly at the uterine fundus; excessive fundal pressure; short umbilical cord; congenital weakness or uterine anomalies; Ehlers-Danlos syndrome and fetal macrosomia. Primiparity as well as rapid labour and delivery are also possible predisposing factors. Some studies have also hypothesized antepartum use of magnesium sulphate and oxytocin as risk factors for uterine inversion however they are yet to be proven scientifically (1, 2).

The clinical presentation of uterine inversion differs according to its degree and timing. Incomplete uterine inversion may be subtle clinically while complete inversion often presents with profuse vaginal bleeding, inability to palpate the fundus abdominally and maternal hemodynamic instability. Uterine inversion may occur before or after placental detachment. Clinical diagnosis is made using bimanual examination wherein the uterine fundus is palpated in the lower uterine segment or within the vagina. Ultrasonography is useful to confirm the diagnosis when clinical examination is suspicious but unclear (2).

***Treatment:*** Fluid resuscitation and control of hemorrhage to restore maternal hemodynamic stability is the main goal of treatment to prevent fatal outcomes. Initial management must focus on reversing the uterus immediately. Uterus reversal can be performed called the Johnson maneuver wherein manual pressure is applied on the fundus through the vagina. This maneuver must be carried out as soon as possible to minimize blood loss and increase chances of resolution since the longer the time between inversion and maneuver, the success rate reduces. This reduction in success rate is due to the involution of the cervix which induces a rigid ring that makes restoration of normal uterus position difficult (3).

Other therapeutic intervention include suspension of oxytocin infusion and administration of utero-relaxant drugs. Magnesium sulphate, terbutaline and salbutamol are most commonly prescribed due to their availability and frequent use. Terbutaline takes about 2 minutes to take effect while magnesium sulphate takes about 10 minutes to be effective. Some authors have reported good outcomes with nitroglycerin (50 to 500 µg) for cervical ring relaxation (3). When tocolytic agents fail to provide uterine relaxation, general anesthesia with halothane, isoflurance, desflurane and sevoflurane may be induced as they are excellent tocolytics. This is particularly useful in patients with hemodynamic instability due to its fewer effects on hemodynamics (4).

***Hydrostatic Reduction:*** Hydrostatic reduction was originally described by O’Sullivan in 1945 wherein pressure of warm fluid infused into the vagina was used to achieve reduction. Other reports include the use of balloons placed intravaginally to increase pressure on the uterine fundus to push it to its original position. Some authors have described its use as an alternative when manual reduction is unsuccesful and conditions for surgical intervention are absent. The use of obstetric vacuum has also been reported to reverse the uterine fundus (3).

***Surgical Management:*** When conservative management fails, it is essential to perform a surgical intervention. Several techniques have been described in existing literature – Huntington, Haultaim, Spinelli and laparoscopic with the first 2 most commonly reported (3). Till date the low incidence of uterine inversion has made no cohort studies large enough to establish the success rate of these techniques. Rarely as a life saving measure peripartum or obstetric hysterectomy maybe performed to achieve control of hemorrhage. 

After repositioning of the uterus administration of uterotonic agents (oxytocin or misoprostol) is essential to prevent recurrence. Broad spectrum antibiotic prescription is also recommended to prevent endometritis or sepsis. In this patient, balloon tamponade was performed after manual reduction and the use of uterotonics which was successfully able to stop the ongoing hemorrhage. 

## Conclusion

Total Uterine Inversion is a rare but potentially deadly complication post vaginal delivery. Its low incidence leads to sparse experience among health professionals in managing this obstetrical emergency. Early fluid resuscitation, manual reposition and balloon tamponade is essential in order to obtain the best prognosis.
